# Measuring health-related quality of life in chronic otitis media in a Chinese population: cultural adaption and validation of the Zurich Chronic Middle Ear Inventory (ZCMEI-21-Chn)

**DOI:** 10.1186/s12955-020-01461-6

**Published:** 2020-07-08

**Authors:** Ruizhe Yang, Ying Zhang, Weiju Han, Yi Li, Shan Li, Jia Ke, Yu Song, Junxiu Liu, Christof Röösli, Alexander M. Huber, David Bächinger, Furong Ma

**Affiliations:** 1 Department of Otolaryngology - Head and Neck Surgery, Peking University Third Hospital, Beijing, 100191 PR China; 2 Center for Clinical Epidemiology and Evidence-based Medicine, Beijing Children’s Hospital, Capital Medical University, National Center for Children’s Health, Beijing, 100045 PR China; 3 Department of Otorhinolaryngology Head and Neck Surgery, Chinese People’s Liberation Army General Hospital, Beijing, 100853 China; 4 Department of Otorhinolaryngology, Tongren Hospital, Capital Medical University, Beijing, 100730 PR China; 5 Department of Otorhinolaryngology, Head and Neck Surgery, University Hospital Zurich, Zurich, Switzerland; 6 University of Zurich, Zurich, Switzerland

**Keywords:** Chronic otitis media, Cholesteatoma, Health-related quality of life, Patient-reported outcome measurement, ZCMEI-21, Chinese

## Abstract

**Background:**

The demand for assessing health-related quality of life (HRQoL) in chronic otitis media (COM) is increasing globally. The currently available Chinese-language patient-reported outcome measurement (PROM) specific for COM includes merely a limited range of related symptoms and dimensions. Hence, in this study, we aim to translate, culturally adapt, and validate the Zurich Chronic Middle Ear Inventory (ZCMEI-21) in Chinese, to enable a comprehensive evaluation of the patients’ subjective health outcome in COM.

**Methods:**

We sampled and surveyed 223 COM patients at three tertiary referral centers in China, using the Chinese translation of ZCMEI-21 (ZCMEI-21-Chn) and the EQ-5D questionnaire, a generic measure of HRQoL. Confirmatory factor analysis (CFA) was performed to investigate the structural model fit to the dataset. Cronbach’s α and test-retest reliability coefficient were calculated to establish reliability, and correlation was tested between ZCMEI-Chn scores and EQ-5D scores for convergent validity.

**Results:**

A total of 208 adult patients with COM were included, with a mean age of 46 years (SD 14 years) and a male proportion of 41% (85/208). A modified bifactor model with ω_H_ of 0.65 and ECV of 0.47 was found to fit the scale scores, indicating fair general factor saturation and multidimensionality of the instrument. ZCMEI-21-Chn demonstrated good reliability (Cronbach’s α = 0.88, test-retest reliability = 0.88). The total scores of ZCMEI-21-Chn had a moderate correlation with a question directly addressing HRQoL (*r* = 0.40, *p* < 0.001), EQ-5D descriptive system score (*r* = 0.57, *p* < 0.001), and EQ-5D visual analogous scale (*r* = 0.30, *p* < 0.001).

**Conclusions:**

The ZCMEI-21-Chn is valid, reliable and culturally adapted to Chinese adult patients with COM. This study offers clinicians an efficient and comprehensive instrument to quantify COM patients’ self-reported health outcomes, which could facilitate the standardization of HRQoL data aggregation in COM on a global scale.

## Background

Chronic otitis media (COM) is a long-term inflammation of the middle ear and mastoid air cells, characterized by recurrent purulent discharge through the tympanic perforation. According to the WHO [1], China has a prevalence of COM ranging from 0.5 to 4%. A significant proportion of the population developed chronic condition as a sequel to acute otitis media (AOM) in their early childhood, and thus suffered from prolonged and cumulative impacts of the disease [2].

COM not only afflicts patients with recurrent or unremitting ear symptoms, including aural drainage, varying degree of hearing impairment etc., but also affects their mental state by generating anxiety or even social alienation [3]. Disturbed health-related quality of life (HRQoL) often complicate patients’ perspectives on the treatment outcome, which may not necessarily be consistent with the physicians' [4]. Therefore, patients’ participation in evaluation should be valued as important supplements to physicians’ viewpoints and physiological evidence. To study HRQoL, patient-reported outcome measures (PROMs) are often used as the assessment tools. These validated questionnaires with clinimetric and psychometric paradigms allow direct and comprehensive descriptions from the patients on their health conditions, and can be applied in both clinical practice and research settings [5, 6].

Currently, the Chinese version of the Chronic Ear Survey (CCES) [7] adapted from the original English Chronic Ear Survey (CES) [8] has been validated and applied nationwide as a Chinese-language PROM to evaluate HRQoL of adult patients with COM [9]. This 13-item questionnaire helps physicians to investigate the health consequences and treatment effectiveness in COM cases from three dimensions: i. Activity Restriction, ii. Symptoms and iii. Medical Resources Utilization [8]. The construction and certain items of CES have been referred to by several new instruments [10–12]. However, the CES does not involve any questions regarding the onset of tinnitus or psychological bearings [13], which are common COM patient complaints that might seriously compromise their HRQoL [3]. Recently, the Zurich Chronic Middle Ear Inventory (ZCMEI-21) [10] has been developed, which is a new questionnaire enabling a comprehensive evaluation of HRQoL. The ZCMEI-21 is subcategorized into four subscales: i. Ear Signs and Symptoms, ii. Hearing, iii. Psychosocial Impact, and iv. Medical Resources. Each subscale contains questions evaluating somatic or psychosocial outcomes scaling from 0 (absence) to 4 (extreme severity).

There has been an increasing need for a universal, disease-specific PROM by worldwide researchers [13–15]. Instruments applicable to different cultural settings allow for consistent trans-national data compilation and comparison. To standardize the reporting of quality-of-life outcomes in COM, the original German ZCMEI-21 has already been adapted into Japanese [16], English [17] and Italian versions [18]. The aims of this present study are to translate the ZCMEI-21 into Chinese (ZCMEI-21-Chn) and validate the Chinese-language instrument in the cultural context.

## Methods

### Patients and study centers

**Fig. 1:**
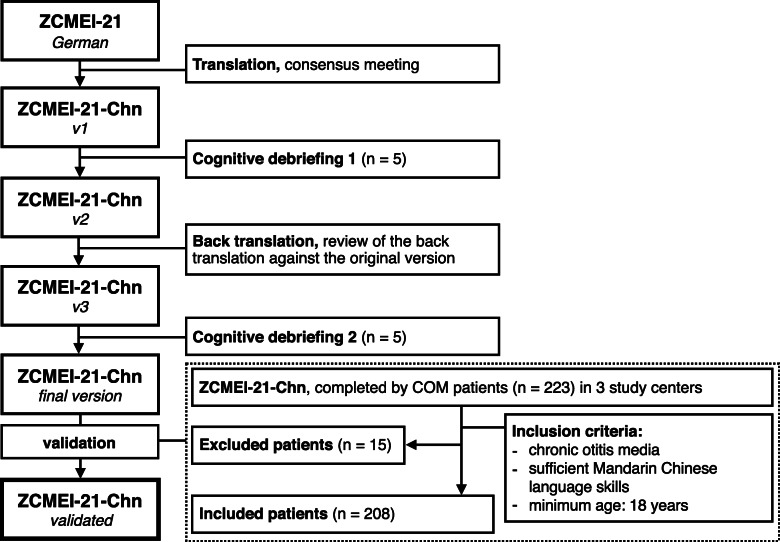
Study design for the translation and validation process of ZCMEI-21-Chn

Inclusion criteria were i. diagnosis of COM with or without cholesteatoma (otitis media chronica cholesteatomatosa [OMCC] and/or otitis media chronica simplex [OMCS]), ii. adult age, iii. Mandarin Chinese as native language. A total of 223 patients were recruited via convenience sampling to complete the ZCMEI-21-Chn, along with the EQ-5D-5L questionnaire, during their outpatient visits at three referral centers between November 2018 and January 2019. Of the 223, 15 were excluded due to missing data, which leaves a total of 208 (93.3%) patients included in this present study (Department of Otolaryngology - Head and Neck Surgery, Peking University Third Hospital, Beijing, PR China: *n* = 61; Department of Otorhinolaryngology Head and Neck Surgery, People’s Liberty Army General Hospital, Beijing, PR China: *n* = 71; Department of Otorhinolaryngology, Tongren Hospital, Beijing, PR China: *n* = 76). The detailed study design and inclusion criteria are described in Fig. 1.

### Translation process

By referring to the ISPOR Principles of Good Practice [19], we standardized the translation and cultural adaption process into the forward, backward, and pretest steps, similar to the procedure of other ZCMEI-21 validation studies (see Fig. 1). The original inventory was primarily translated by two authorized translators independently. A native Chinese-speaking otologist with high proficiency in German then revised and merged these two transcriptions into a reconciled version, ZCMEI-21-Chn v1. A pilot test on v1 was conducted by 5 subjects, followed by cognitive debriefing and consensus meeting with the development team, though not explicitly recommended by ISPOR. V1 was then modified into ZCMEI-21-Chn v2 based on the feedbacks from the respondents. A third professional translation agency with medical background later translated ZCMEI-21-Chn v2 back into German. Certain items have been culturally adapted in the back translated version. For instance, on noticing that a significant proportion of the respondents were unable to connect their experience of dizziness with “the loss of balance control” originally stated in Question 5, we altered the description of this item into “Have you been experiencing dizziness or loss of balance?”. The transcription was reviewed against the original German version and revised for minor differences, before being subjected to a second cognitive debriefing process on another 5 subjects with no further adjustments. The final version of ZCMEI-21-Chn used in the following validation process was provided in paper-based form.

### Validation process

#### Questionnaire survey

The final version of ZCMEI-21-Chn, along with the 5-level EuroQol five-dimensional questionnaire (EQ-5D-5L [20], referred to as EQ-5D), was administrated by patients meeting the abovementioned inclusion criteria at the clinics. Concurrent audiometric data were obtained from all the recruited cases. The layout panel from the original script [10] was adopted in the Chinese version of ZCMEI-21. For validation purposes, we involved an extra question directly assessing the general quality of life (Question 22, “My ear illness is worsening my quality of life…not at all/ mildly/ moderately/ severely/ very severely”).

The EQ-5D is a preference-based instrument used world-wide [21, 22] to assess generic HRQoL [23], and it comprises of the EQ-5D descriptive system and the EQ Visual Analogue Scale (EQ VAS). The former defines health in terms of 5 dimensions: i. Mobility, ii. Self-Care, iii. Usual Activities, iv. Pain/Discomfort and v. Anxiety/Depression; each dimension is depicted at five levels, corresponding to no, slight, moderate, severe and extreme problems. The latter, EQ VAS, measures the self-rated health state on the day of interview, ranging from 0 to 100 (corresponding to “worst” to “best imaginable health state”). The Chinese version of the EQ-5D questionnaire, administrated in the validation study, has been validated with a full set of rescaled EQ descriptive system scores [24], scaling from − 0.391 to 1.

The role that cultural differences play in shaping the patients’ perception of items originally developed in a foreign background was taken into consideration during the process. In addition, the results of the other international validation studies on the adapted versions of ZCMEI-21 enlightened us to hypothesize the correlation between the generic HRQoL scores and the COM-specific scale scores as moderate and positive.

Quality control was performed throughout the present study. Participants were provided with detailed verbal and written instruction on scale filling if needed. Unified training for standard procedure of recording of the questionnaires was conducted among all researchers. Each questionnaire was double entered and was checked in time.

### Statistical analysis

Statistical analysis was performed using SAS (version 9.4, SAS Institute Inc., Cary, NC, USA), *R* Software (version 3.6.3, The *R* Foundation for Statistical Compupting), Mplus (version 7.4, Muthén & Muthén, CA, USA) and GraphPad Software (version 8, GraphPad Software, La Jolla, CA, USA). A two-tailed p-value less than 0.05 was considered statistically significant for all analyses. Values were reported as mean (SD) or as absolute number and percentage. The frequency distribution of the ZCMEI-21-Chn total scores was inspected through both a graphical approach and a normality test. The bell-shaped distribution fitting a normal probability curve in the histogram, and a p > 0.05 in D’Agostino-Pearson normality test indicated Gaussian distribution of the data. Items with a value of item total-correlation (ITC) corrected for overlap ≥0.3 were deemed as a “strong item” [10].

Before structural detection, sampling adequacy was confirmed via the Kaiser-Meyer-Olkin (KMO) test and Bartlett’s test of sphericity. A KMO value ≥0.80 and a *p* < 0.05 in Bartlett’s test indicated suitability of the data for factorial analysis. The developer of ZCMEI-21 suggested a hypothesized structure model comprising of 4 dimensions that also supported an overall score of the scale [10]. Therefore, a bifactor model and a second-order model was examined through fit indexes via confirmatory factor analysis (CFA) [25]. Based on theoretical considerations and statistical indications, models were modified to acquire fitting solution. Cutoff levels of fit indexes were: Root Mean Square Error of Approximation (RMSEA) < 0.06; both Comparative Fit Index (CFI) and Non-Normed Fit Index (NNFI), also known as Tuker-Lewis Index (TLI) > 0.95 [26]. Modified model fit was reanalyzed to prove statistical superiority over the original model via a chi-square difference test and comparison of AIC and BIC. Coefficient omega hierarchical (ω_H_) and explained common variance (ECV) were calculated to estimate the proportion of variance attributable to the single general target trait (general factor, G) [27], and to measure the unidimensionality of the scale [25] accordingly.

The pure-tone average (PTA) at speech frequency (0.5 kHz, 1 kHz, 2 kHz, and 4 kHz) [28] were collected from the patient’s concurrent audiometry testing. Criteria validity of ZCMEI-21-Chn was assessed with correlations to the PTAs of worse- and better-hearing ear.

Convergent validity was established by studying correlation between the total ZCMEI-21-Chn s cores, the additional question (Question 22) that directly addressed HRQoL the and EQ-5D descriptive system and VAS scores were examined using Pearson’s correlation analysis. Cronbach’s α and test-retest reliability suggested the reliability of the ZCMEI-21-Chn, with acceptance range set to ≥ 0.70 [7, 29].

The sample size for the validation survey was determined based on a subject to item ratio of 10:1, i.e. 21*10 = 210 cases [30].

## Results

Detailed characteristics of the 208 respondents are listed in Table 1. Questions 8–10 assessing hearing impairment in detail were automatically skipped by 25 patients [10], who claimed, in Question 7, no detectable hearing impairment within the last 2 weeks.

**Table 1:** Patients Characteristics of the Validation Cohort

	Validation Cohort (*n* = 208)
Male to female ratio	85 (41%): 123 (59%)
Age (years)	46 (14)
COM, type - n%	
- OMCS	150 (72%)
- OMCC	51 (25%)
- both	7 (3%)
Affected Ear(s)	
- left	92 (44%)
- right	70 (34%)
- both	46 (22%)
Previous surgical history due to COM – n%	
yes	37 (18%)
no	171 (82%)
Disease duration (months)	221 (203)

Data are mean (SD) or number (%)

*COM* chronic otitis media; *OMCS* otitis media chronica simplex; *OMCC* otitis media chronica cholesteatomatosa

Single item statistics showed well-distributed answers and full range of answers (0–4) in every question. Detailed descriptive statistics of the individual items and subscales are listed in Table 2. The Item-Total-Correlation (ITC), corrected for overlap with the scale total, as one of the criteria for a strong item, was above 0.3 for all items except question 5 [10]. Correlation among the four subscales ranges from 0.18 to 0.61 (*p* < 0.001). Each subscore was moderately to strongly correlated with the ZCMEI-21-Chn total scores (see Table 3).

**Table 2:** Descriptive statistics of the individual items (ZCMEI-21-Chn)

Number	Subscale/Item	Mean	Min-Max	ITC (adjusted)	Cronbach’s α
I.	Ear Symptoms and Signs				0.60
1.	Ear Pain	0.81	0–4	0.30	
2.	Discharge	1.80	0–4	0.30	
3.	Itching	1.27	0–4	0.42	
4.	Feeling of Pressure	1.68	0–4	0.43	
5.	Dizziness	0.32	0–4	0.26	
II.	Hearing				0.77
6.	Tinnitus	1.63	0–4	0.32	
7.	Hearing (filter question)	2.47	0–4	0.47	
8.	When many people speak at the same time	1.80	0–4	0.57	
9.	Telephone, alarm clock	1.00	0–4	0.50	
10.	Fear of not hearing other people	1.77	0–4	0.55	
III.	Psychosocial Impact				0.86
11.	Impact of ear symptoms on HRQoL	2.35	0–4	0.64	
12.	Activities with family and friends	1.21	0–4	0.64	
13.	In public (e.g. school/occupation, shopping)	1.39	0–4	0.73	
14.	Making contact with other people	1.45	0–4	0.70	
15.	Quality of sleep	1.35	0–4	0.60	
16.	Sadness	1.63	0–4	0.62	
17.	Fear that the ear problems may persist	2.31	0–4	0.50	
18.	Protection from water	1.74	0–4	0.27	
IV.	Medical Resources				0.71
19.	Medical consultations	1.46	0–4	0.31	
20.	Antibiotics (oral)	1.11	0–4	0.32	
21.	Ear drops	1.41	0–4	0.37	

*ITC* Item-Total-Correlation

*Cronbach’s α* for individual subscales

**Table 3:** Descriptive statistics and correlation of subscales and total scores of ZCMEI-21-Chn

	D1	D2	D3	D4
Items involved	Question 1–5	Question 6–10	Question 11–18	Question 19–21
Mean	5.88 (3.80)	8.67 (4.77)	13.42 (7.74)	3.98 (2.56)
Correlation to D2	*r* = 0.30 ^a^			
Correlation to D3	*r* = 0.44 ^a^	*r* = 0.61 ^a^		
Correlation to D4	*r* = 0.37 ^a^	*r* = 0.18 ^a^	*r* = 0.32 ^b^	
Correlation Coefficient to ZCMEI-21-Chn total scores	*r* = 0.66 ^b^	*r* = 0.77 ^b^	*r* = 0.91 ^b^	*r* = 0.51^b^

*ZCMEI-21-Chn* Chinese version of Zurich Chronic Middle Ear Inventory

^a^ Correlation coefficients were significant at *p* < 0.001, ^b^ Correlation coefficients were significant at *p* < 0.0001

**Fig. 2:**
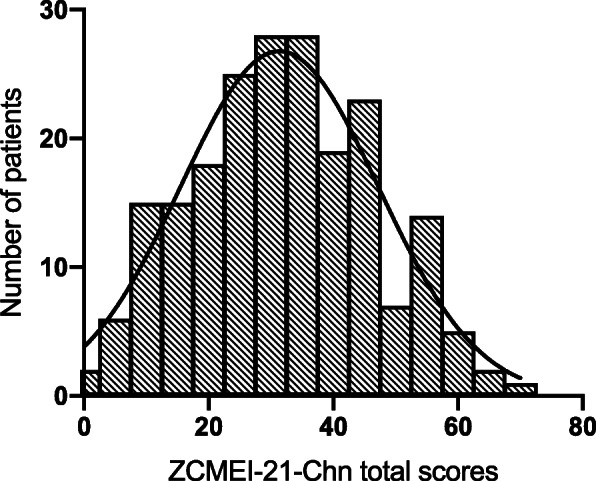
ZCMEI-21-Chn total scores distribution and best-fitting Gaussian curve (bin-width on x-axis: 5)

**Fig. 3:**
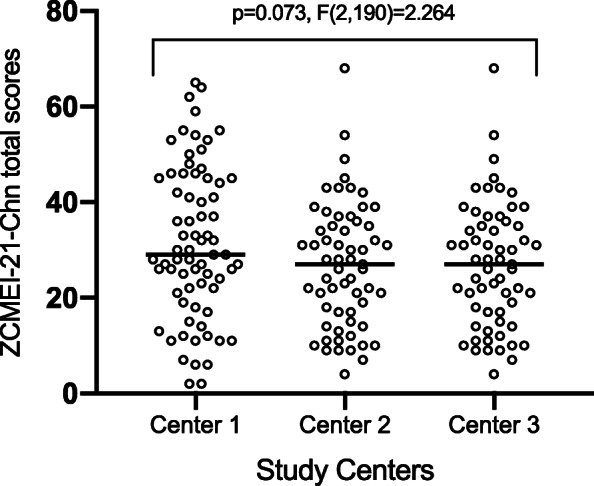
ZCMEI-21-Chn total scores at the three referral centers

ZCMEI-21-Chn total scores followed a Gaussian distribution, suggested by both the histogram (Fig. 2) and the D’Agostino & Pearson normality test (*p* = 0.06). No significant differences of ZCMEI-21-Chn total scores among the three study centers were observed (*p* = 0.07, F(2,190) = 2.644, one-way ANOVA; Fig. 3).

**Fig. 4:**
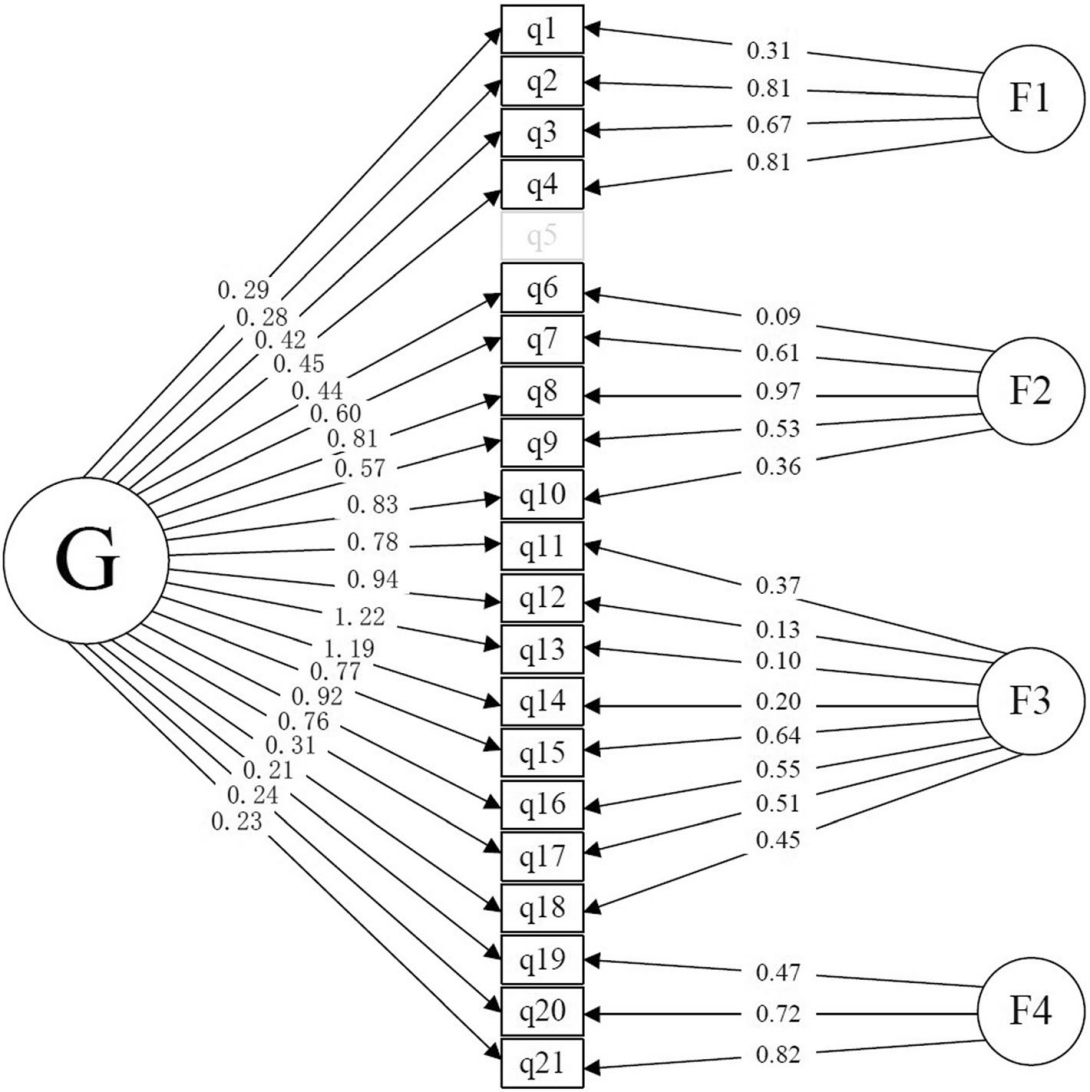
Modified bifactor model that best fits the data

By conducting the KMO test and Bartlett’s test of sphericity on the data obtained, we found a KMO value of 0.87 and a p-value less than 0.001 respectively. From these results, we confirmed our data to be suitable to construct investigation via factorial analysis. The fit statistics of the hypothesized bifactor model with four domain-specific factors and the corresponding second-order model with four lower-order factors were reported in Table 4. The results of these fit statistics indicated post-hoc modification to both models. We obtained alternative bifactor and second-order solutions by deleting Question 5. Among all observable variables, item q5 was found with the lowest factor loading to the latent variables, also most poorly understood by the Chinese patient group. The chi-square difference test suggested that the modified bifactor model (Fig. 4) was a statistically better fit (Δχ^2^_(18)_ = 32.96, p < 0.05) [31, 32] than the hypothesized construct. Also, RMSEA, NNFI and CFI of the modified bifactor model all fell within acceptable range, while those of the modified second-order model did not. Coefficient ω_H_ of the general factor in the model fit was 0.65, and ECV was 0.47. For detailed fit statistics and the loading matrix of the hypothesized and the trimmed model, please refer to Tables 4 and 5 and Fig. 4.

**Table 4:** Fit indices of the hypothesized and the modified models

	Models	χ^2^	Δf	RMSEA	NNFI (TLI)	CFI	AIC	AIC Saturated	BIC
Hypothesized	Bifactor model	270.83*	168	0.05	0.94	0.94	394.25	− 1098.42	637.89
	Second-order model	376.02*	185	0.07	0.87	0.88	468.02	− 1099.06	621.55
Modified	Bifactor model	237.25*	150	0.05	0.96	0.95	348.26	− 992.34	581.90
	Second-order model	338.93*	181	0.07	0.89	0.88	426.92	− 1071.04	573.78

χ^2^ chi-square; *Δf* degree of freedom; *RMSEA* Root Mean Square Error of Approximation; *NNFI* Non-Normed Fit Index, also known as *TLI* Tuker-Lewise Index; *CFI* Comparative Fit Index; *AIC* Akaike Information Criteria; *BIC* Bayesian Information Criterion

*: *p* < 0.001

**Table 5:** Factor loadings from the hypothesized and the modified bifactor models of the ZCMEI-21-Chn

		Hypothesized Bifactor Model										Modified Bifactor Model									
Item	Subscale/Item	G		F1		F2		F3		F4		G		F1		F2		F3		F4	
		LE	SE	LE	SE	LE	SE	LE	SE	LE	SE	LE	SE	LE	SE	LE	SE	LE	SE	LE	SE
1.	Ear Pain	0.29	0.08	0.31	0.11							0.29	0.08	0.31	0.10						
2.	Discharge	0.28	0.10	0.81	0.12							0.28	0.10	0.81	0.12						
3.	Itching	0.42	0.08	0.67	0.11							0.42	0.08	0.67	0.11						
4.	Feeling of Pressure	0.45	0.10	0.81	0.11							0.45	0.09	0.81	0.11						
5.	Dizziness	0.18	0.07	0.06	0.07																
6.	Tinnitus	0.45	0.10			0.08	0.11					0.44	0.09			0.09	0.11				
7.	Hearing (filter question)	0.60	0.10			0.61	0.09					0.60	0.08			0.61	0.09				
8.	When many people speak at the same time	0.81	0.09			0.97	0.10					0.81	0.08			0.97	0.10				
9.	Telephone, alarm clock	0.58	0.10			0.56	0.09					0.57	0.09			0.56	0.09				
10.	Fear of not hearing other people	0.83	0.09			0.35	0.09					0.83	0.09			0.36	0.09				
11.	Impact of ear symptoms on HRQoL	0.78	0.09					0.37	0.11			0.78	0.09					0.37	0.11		
12.	Activities with family and friends	0.94	0.08					0.13	0.13			0.94	0.08					0.13	0.13		
13.	In public (e.g. school/occupation, shopping)	1.22	0.07					0.11	0.13			1.22	0.08					0.10	0.12		
14.	Making contact with other people	1.19	0.07					0.21	0.11			1.19	0.07					0.20	0.11		
15.	Quality of sleep	0.77	0.10					0.63	0.12			0.77	0.10					0.64	0.12		
16.	Sadness	0.92	0.09					0.54	0.14			0.92	0.09					0.55	0.14		
17.	Fear that the ear problems may persist	0.76	0.10					0.51	0.12			0.76	0.09					0.51	0.12		
18.	Protection from water	0.31	0.10					0.44	0.13			0.31	0.10					0.45	0.13		
19.	Medical consultations	0.21	0.07							0.47	0.07	0.21	0.07							0.47	0.07
20.	Antibiotics (oral)	0.24	0.08							0.72	0.10	0.24	0.08							0.72	0.10
21.	Ear drops	0.33	0.09							0.82	0.11	0.33	0.09							0.82	0.11

*G:* general factor*; F1: Factor 1; F2:* Factor 2*; F3:* Factor 3*; F4:* Factor 4

*LE* Factor loading estimates; *SE*: Standard error

Cronbach’s α of ZCMEI-21-Chn was 0.88, with all subscales’ above 0.70, except for the ear symptoms dimension. There were 53 patients who had not undergone significant clinical change from last visit, and were readministrated with the scale after a three- to four-week interval. And the test-retest reliability coefficient was also 0.88.

For validity evaluation, we observed a moderate correlation (*r* = 0.40, *p* < 0.001) between the question directly addressing HRQoL (Question 22) and the total scores of ZCMEI-21-Chn. The EQ descriptive scores were moderately correlated with the ZCMEI-21-Chn total scores (*r* = 0.57, *p* < 0.001). Yet, a weaker correlation was found between EQ-5D VAS and the ZCMEI-21-Chn total scores (*r* = 0.30, *p* < 0.0001). Next, the ZCMEI-21-Chn factor scores were correlated to the EQ-5D scores. Subscale representing the psychosocial impact, was strongly correlated to the EQ-5D descriptive system scores.

The correlation between the ZCMEI-21-Chn and the audiometric data were demonstrated in Table 6. Both worse-hearing ear PTA [53.84 (23.96) dB HL] and better-hearing ear PTA [25.82 (19.44) dB HL] significantly correlated with the hearing-related items and the total scores.

**Table 6:** Correlation between ZCMEI-21-Chn and audiometric data

		Worse-hearing ear PTA		Better-hearing ear PTA	
Dimensions	Hearing-related items	r	p	r	p
6	Tinnitus	0.12	< 0.05	0.14	< 0.05
7	Hearing (filter question)	0.41 *	< 0.05	0.28 *	< 0.05
8	When many people speak at the same time	0.48 *	< 0.05	0.39 *	< 0.05
9	Telephone, alarm clock	0.38 *	< 0.05	0.36 *	< 0.05
10	Fear of not hearing other people	0.32 *	< 0.05	0.31 *	< 0.05
I	Ear signs and symptoms	0.17 *	< 0.05	0.22 *	< 0.05
II	Hearing	0.45 *	< 0.05	0.39 *	< 0.05
III	Psychosocial impact	0.35 *	< 0.05	0.39 *	< 0.05
IV	Medical resources	0.06 *	0.3175	0.09	0.1676
Total score	ZCMEI-21-Chn	0.40 *	< 0.05	0.38 *	< 0.05

*: Correlation coefficients were significant at *p* < 0.05

Lastly, while comparing the total and subscale scores of ZCMEI-21-Chn, and the EQ-5D descriptive system score, a good level of comparability to the respective scores reported in the original validation study was discovered. Table 7 offers an extensive prospect over the global commonalities of the validation process for the various ZCMEI adapted versions.

**Table 7:** Descriptive statistics and correlation of the ZCMEI-21-Chn and the EQ-5D-5L, Cronbach’s α in the present study and the original validation study of the German-language ZCMEI-21, ZCMEI-21-Jap, ZCMEI-21-E, ZCMEI-21-It

	Current Study (ZCMEI-21-Chn)	ZCMEI-21 [10]	ZCMEI-21-Jap [16]	ZCMEI-21-E[17]	ZCMEI-21-It [18]
ZCMEI-21 total score (SD)	32.0 (14.5)	29.7 (16.1)	25.2 (11.8)	25.9 (15.8)	29.8 (14.6)
I. Ear signs and symptoms	5.9 (3.8)	5.1 (3.9)	2.3 (2.0)	4.2 (3.9)	5.5 (4.5)
II. Hearing	8.7 (4.8)	8. 5 (5.2)	7.6 (5.1)	8.2 (5.1)	7.6 (5.1)
III. Psychosocial impact	13.4 (7.7)	13.1 (7.9)	8.2 (6.6)	10.9 (7.9)	13.7 (7.8)
IV. Medical resources	4.0 (2.6)	3.0 (2.3)	3.0 (2.4)	2.6 (2.4)	2.9 (2.7)
EQ-5D-5L (SD)					
Descriptive system score	0.84 (0.17)	0.92 (0.14)	0.89 (0.15)	0.80 (0.23)	0.84 (0.15)
VAS score	77.1 (13.7)	N/A	77.3 (16.6)	79.0 (19.7)	76.6 (15.0)
Total score correlation					
To question directly assessing HRQoL	*r* = 0.40 ^a^	*r* = 0.74 ^a^	*r* = 0.68 ^a^	*r* = 0.74 ^a^	*r* = 0.62 ^a^
To EQ-5D-5L descriptive system score	*r* = 0.57 ^a^	*r* = 0.60 ^a^	*r* = 0.49 ^a^	*r* = 0.60 ^a^	*r* = 0.39 ^a^
Cronbach’s α	0.88	0.91	0.85	0.91	0.86

*ZCMEI-21-Chn* = Chinese version of Zurich Chronic Middle Ear Inventory

*ZCMEI-21-Jap* = Japanese version of Zurich Chronic Middle Ear Inventory

*ZCMEI-21-E* = English version of Zurich Chronic Middle Ear Inventory

*ZCMEI-21-It* = Italian version of Zurich Chronic Middle Ear Inventory

*EQ-5D-5 L* = EuroQol five-dimensional questionnaire in its five-level version

Data are mean (SD)

^a^ Correlation coefficients were significant at *p* < 0.001

## Discussion

In the present study, we translated the ZCMEI-21 into Chinese by following the international guidelines. Next, we validated the ZCMEI-21-Chn in a multi-center study. Cronbach’s α and test-retest reliability coefficient of 0.88 indicated a good level of reliability of the entire questionnaire. Despite a lack of clear recommendations for the translated versions of an established scale, α ≥ 0.70 is a commonly used level for reliable measure in population studies [7]. With sampling adequacy to factor analysis confirmed with KMO and Barlett’s test, CFA was performed to seek structural models that fit the data. The results of CFA suggested that the modified bifactor model provided a significantly better fit to the matrix than the original hypothesized models. An ω_H_ of 0.65 provided quantitative evidence that the scale scores generalize to a relative high extent to a latent variable (general factor, G) common to all the direct variables (except item 5). However, the domain-specific factors account for 53% (1-ECV) of the total variance, indicating that both an overall scoring and a combination of the subscores are meaningful interpretations of the scale [33].

Within CFA, the fifth item (q5) that questioned the patients about their feelings of dizziness or loss of balance control was already revised for inexplicability during the prior translation stage. The revised q5 turned out to remain poorly understood with the lowest loading to both F1(the symptom dimension, 0.06) and G(general factor, 0.18). The fit statistics of the model with complete deletion of q5 excels the one that only removed q5 from F1. Meanwhile, loading on item 6 (tinnitus) to F2 (hearing dimension) was 0.08, and 0.45 to G in the original bifactor model. We have also tried to modify the model by subtracting q6 from F2 solely or in combination with deleting q5. However, the fit indices of both models were found unacceptable, which suggested that further post-hoc modification would make neither theoretical nor statistical sense.

Furthermore, item 18 (protection from water)‘s loading to G was lower than the other items included in the psychsocial dimension (F3). Consistent with the findings during survey, the daily water-proof precautionary measures seemed to bother Chinese COM patients far less than the inconvenience brought by ear drainage or hearing impairment. Quite commonly did these patients respond to item 18 with “I have got used to wearing these earplugs during showers” or “It’s fine that I have quitted swimming ever since the onset of the disease”. Such discoveries that may involve a role of cultural ambiance inspire the interests and efforts in our future studies.

In addition, the fourth dimension (medical resources) demonstrated good internal reliability, yet showed the weakest correlation to both ZCMEI-21-Chn total scores and EQ-5D scores, as well as a non-significant correlation to PTA of either better- or worse-hearing ear. On one hand, this may suggest that seeking treatment played a comparatively limited part in hampering the general HRQoL in Chinese COM patients; on the other hand, the frequency of clinic appointments and medicine usages might not only be affected by the severity of hearing impairment or other complications, but also by the uneven distribution and accessibility of medical resources across the country.

A moderate correlation between total scores and the question directly addressing HRQoL might be explained by the underlying variance in perception and requirement of living standard in the Chinese cultural background. Weak to moderate correlation between the ZCMEI-21-Chn total, subscale scores and EQ-5D scores were well within expectation, since generic PROMs are reportedly less sensitive to self-rated HRQoL than disease-specific measures in hearing impaired or COM patients [34, 35]. Noticeably, correlation to the ZCMEI-21-Chn total and subscale scores of the EQ VAS scores were overall weaker than that of the EQ-5D descriptive system scores, possibly implying that the Chinese patients were unfamiliar with the visual analogous scale for the use of rating their health state.

Although no statistical comparison was performed, qualitative comparison revealed that the ZCMEI-21-Chn scored higher both overall and in each subset than the original German-language ZCMEI-21, as well as the other international adapted versions. This might be attributable to the relative scarcity of the primary care accessible to the Mandarin speaking population. In which case, only patients in a rather progressive state of the disease would come to seek professional help at the tertiary referral center in the capital. Thus, to assess the baseline ZCMEI-21-Chn scores of the Chinese COM population in order to compare with the other countries’, requires larger-scaled and more representative sampling in future studies.

There are a number of limitations in this study that ought to be acknowledged. First, EQ-5D was applied to the testing of convergent validity of ZCMEI-21-Chn, yet none to studying the hetrerotrait correlations in discriminant validity. Nonetheless, to administrate extra questionnaires during clinical visits at China’s overloaded tertiary referral centers seems infeasible. Future efforts will be devoted to issuing extended versions of ZCMEI-21-Chn applicable on the electric devices to allow comprehensive assessment and better experience of the patients.

The assumptions, under which Cronbach’s α is a consistent estimator for reliability, may not be entirely attainable in this study. For example, the varying factor loadings in the model fits were contrary to *tau* equivalence. Correlated error might arise from the order of items on the scale, speeded tests and so on [36]. Moreover, the multidimensionality and the Pearson correlation matrix may also bias the estimates of Cronbach’s α. On account of these unrealistic assumptions, a 5-level Likert, multidimensional scale like ZCMEI-21-Chn, may require substitute indicators for internal consistency, e.g. McDonald’s Omega or coefficients in G-Theory in future researches. Cronbach’s α was kept in the present study, also for the parametric comparison with the other international versions of ZCMEI-21.

Another limitation to our study is that, without different item functioning (DIF), the statistical results reported in this article may only serve as a rough guide to measure the relationship of this current study with the original research. Additionally, the sampling was neither randomized nor representative, rather purposive, which possibly resulted in the heterogeneity of the subjects, e.g. in ethnicity or education level. To further adapt ZCMEI-21-Chn, additional studies are needed focusing on balancing the potential ethnic influence.

## Conclusion

We translated and culturally adapted the ZCMEI-21 into Chinese, and demonstrated the ZCMEI-21-Chn to be a reliable and valid self-reported outcome measure. Scores of the entire scale as well as of each dimension can be used to evaluate HRQoL in adult Chinese patients with COM. With health professionals’ understanding of the disease impacts and treatment effectiveness deepened, our future efforts include implementing the electronic version of ZCMEI-21-Chn as clinical routine, and enhancing standardized data aggregation on a global scale.

## Supplementary information

**Additional file 1.**

## Data Availability

The dataset used and/or analyzed in this current study are available from the corresponding authors on reasonable request. The Chinese version of Zurich Chronic Middle Ear Inventory (ZCMEI-21-Chn) is included in the supplementary information file.

## Abbreviations

AOM: Acute otitis media
CCES: Chinese version of chronic ear survey
CES: Chronic ear survey
COM: Chronic otitis media
EQ-5D: European quality of life-5 dimensions questionnaire
HRQoL: Health-related quality of life
ITC: Item total correlation
max: Maximum
min: Minimum
OMCC: Otitis media chronica cholesteatomatosa
OMCS: Otitis media chronica simplex
PROM: Patient-reported outcome measurement
SD: Standard deviation
CFA: Confirmatory factor analysis
ZCMEI-21: Zurich chronic middle ear inventory
ZCMEI-21-Chn: Chinese version of Zurich chronic middle ear inventory
ZCMEI-21-E: English version of Zurich chronic middle ear inventory
ZCMEI-21-It: Italian version of Zurich chronic middle ear inventory
ZCMEI-21-Jap: Japanese version of Zurich chronic middle ear inventory
