# Supplementary material for: Measuring health-related quality of life in chronic otitis media in a Chinese population: cultural adaption and validation of the Zurich Chronic Middle Ear Inventory (ZCMEI-21-Chn)
Source: Health Qual Life Outcomes. 2020 Jul 8;18:218. doi: 10.1186/s12955-020-01461-6 (PMC7346638; doi:10.1186/s12955-020-01461-6)
Supplement: Supplementary file 1 — Additional file 1. [file 12955_2020_1461_MOESM1_ESM.pdf]

慢性中耳炎患者生活质量调查问卷（苏黎世中耳炎量表中文版，ZCMEI-21-Chn）

请评估您**过去两周内**以下症状的严重程度:

	无此症状	症状轻微	症状一般	症状明显	非常严重
1. 耳痛	○	○	○	○	○
2. 耳中有液体流出	○	○	○	○	○
3. 耳内瘙痒	○	○	○	○	○
4. 耳中有闷堵感	○	○	○	○	○
5. 头晕或难以维持平衡	○	○	○	○	○

	完全没有	偶尔有	有时有	较为严重	非常严重
6. 过去的两周内我有耳鸣	○	○	○	○	○

	听力很好	基本没问题	有时听不清	经常听不清	一直听不清
	**请跳转至第 11 题**		**请继续回答第 8 题**		
7. 过去的两周内我的听力很好	○	○	○	○	○

	听力很好	基本没问题	有时听不清	经常听不清	一直听不清
8. 我在嘈杂环境中也能听清并理解别人说的话	○	○	○	○	○
9. 我接听电话和听清闹钟没有问题	○	○	○	○	○

	完全不符合	不太符合	部分符合	基本符合	完全符合
10. 我时常担心无法理解他人说的话	○	○	○	○	○
11. 耳朵的症状给我造成了不小的心理负担（例如耳痛、耳中流出液体、耳闷堵感、听力下降、耳鸣或/及其他）	○	○	○	○	○
12. 耳朵的问题导致我和家人交谈减少	○	○	○	○	○
13. 由于耳朵的问题，我在上学/工作、或在其他公共场合活动（例如购物）都受到限制	○	○	○	○	○
14. 耳朵的问题影响我同他人的交往	○	○	○	○	○
15. 耳朵的问题影响我的睡眠质量	○	○	○	○	○
16. 耳朵的问题让我情绪低落	○	○	○	○	○
17. 我担心耳朵的问题以后也无法改善	○	○	○	○	○

	没有不便	稍有不便	较为不便	很不方便	非常不便
18. 请问防止耳朵进水是否为您带来很大的不便？<br>如果您不需要防止耳朵进水，请选择“没有不便”	○	○	○	○	○

	1 次	2-3 次	4-7 次	8-15 次	16 次及以上
19. 在**过去的 6 个月中**，您因为耳朵问题去就医的次数？	○	○	○	○	○

	没有使用	数天	数周	数月	一直在用
20. 您在**过去的 6 个月中**，因为耳部疾病使用抗菌药物（头孢、克拉霉素、阿莫西林、阿奇霉素等）治疗的总时长为？	○	○	○	○	○
21. 您在**过去的 6 个月中**，因为耳部疾病使用滴耳液的总时长为？	○	○	○	○	○
